# Protecting public health and the environment: towards a general ban on cellulose acetate cigarette filters in the European Union

**DOI:** 10.3389/fpubh.2023.1282655

**Published:** 2023-10-31

**Authors:** Stijn Everaert, Greet Schoeters, Filip Lardon, Annelies Janssens, Nicolas Van Larebeke, Jean-Marie Raquez, Lieven Bervoets, Pieter Spanoghe

**Affiliations:** ^1^Chemical Environmental Factors Group, Superior Health Council, Brussels, Belgium; ^2^Department of Biomedical Sciences, University of Antwerp, Antwerp, Belgium; ^3^Center for Oncological Research (CORE), University of Antwerp, Antwerp, Belgium; ^4^Department of Thoracic Oncology, University Hospital Antwerp, Antwerp, Belgium; ^5^Department of Radiotherapy and Experimental Cancerology, Ghent University, Ghent, Belgium; ^6^Department of Analytical, Environmental and Geo-Chemistry, Vrije Universiteit Brussel, Brussels, Belgium; ^7^Polymer and Composite Materials Department, University of Mons, Mons, Belgium; ^8^Department of Biology, University of Antwerp, Antwerp, Belgium; ^9^Faculty of Bioscience Engineering, Ghent University, Ghent, Belgium

**Keywords:** smoking prevention, tobacco, cigarette filter, adenocarcinoma, ecotoxicology

## Abstract

After the establishment of a causal relationship between tobacco use and cancer in the 1950s, cellulose acetate filters were introduced with the claim to reduce the adverse health impact of unfiltered cigarettes. Often perceived to be more pleasant and healthy, filters encouraged smoking. However, filtered cigarettes are more deeply inhaled to obtain the same nicotine demand while altered combustion releases more tobacco-specific nitrosamines. The increasing use of cigarette filter ventilation is associated with a sharp rise in lung adenocarcinomas in recent decades. While not preventing adverse health effects, a global environmental problem has been created due to the non-biodegradable filter litter, causing ecotoxicological effects and the spread of microplastics. Recently, the Belgian Superior Health Council advised policymakers to ban cigarette filters as single-use plastics at both national and European levels. This article outlines the arguments used to justify this plea (human health and environment), the expected effects of a filter ban, as well as the public reception and reactions of the tobacco industry. The specific context of the European Union is discussed including the revision of the Single-Use Plastics Directive, affording a new opportunity to ban plastic filters. This perspective article aims to fuel the momentum and cooperation among member states for this purpose.

## 1. Introduction

Since the 1950s, a causal association has been established between tobacco smoking and lung cancer (1–3). Besides the presence of about 9,500 chemicals in cigarette smoke, 83 different IARC-classified carcinogens have been identified in unburned tobacco and tobacco smoke (4). A main response of the tobacco industry was the introduction of filtered cigarettes (with or without ventilation holes), although the history of the filter goes further back up to the 1860s (5, 6). As filters can reduce tar, nicotine, and carbon monoxide (TNCO) intake per cigarette and particle concentrations (7–10), the industry actively promoted the idea that filters reduce health risks for smokers (11). This resulted in a false perception of greater safety among smokers of filtered, so-called “light” and “ultra-light” cigarettes (12–14). During the past three decades, the benefits of filters were disputed by many researchers and the WHO, with pleas for a filter ban growing louder (5, 6, 15–19). Moreover, controversy recently arose in the Netherlands about the presence of filter ventilation holes that dilute mainstream smoke. Due to their presence, standard ISO 3308 smoke machines used to assess cigarette emissions strongly underestimate the actual exposure of smokers to TNCO and aldehydes (20–22). This led to the Dutch term “*sjoemelsigaret*” (fraudulent cigarette), as the underestimation of the ISO method was formally affirmed by the Court of Rotterdam on November 4th, 2022 (23). In the context of these developments, the Belgian Minister of Environment asked an interdisciplinary working group of the Superior Health Council (SHC) for advice, which was published in April 2023, advocating a European ban on cellulose acetate filters (24). This position was supported by a broad front of national medical, paramedical, and patient organizations, and received wide coverage in Belgian media.

In this perspective article, it is aimed (1) to provide a scientific state-of-the-art of health and environmental arguments, (2) to discuss the expected effects of a filter ban, (3) to illustrate the reception of the Belgian initiative including reactions of the tobacco industry and (4) to discuss the specificity of the European institutional context for a filter ban, along with the next opportunity.

## 2. The health perspective

Given that filter use only increased exponentially since the 1950s and mid-1960s, the health effects of filters were poorly understood during the 20th century. This was complicated by lag times of lung cancer and possible epidemiological selection bias (e.g., sociological differences, smoking history and intensity). In 1986, the International Agency for Research on Cancer (Vol. 38) noted that some case–control and cohort studies (25–29) suggested greater risks for prolonged use of nonfilter and “high-tar” cigarettes (30). However, the IARC refrained from drawing premature conclusions. Due to the reduced particle numbers and TNCO per cigarette, filtered cigarettes are often perceived to be less harmful (12, 13). However, health issues should not be viewed on cigarette scale but as a function of individual nicotine demand. In 1989, Augustine et al. (31) noted that switching to filtered cigarettes may induce compensation behavior to meet the personal nicotine demand, increasing the total number of cigarettes smoked per day. Moreover, as filtered cigarettes reduce irritation, taste more pleasant and are perceived healthier, filters encourage people to smoke more cigarettes per day (12, 16). Compensation is indeed affirmed by human biomonitoring. When the number of cigarettes is taken into account, smoking-machine derived carbon monoxide (CO) and cyanide (CN) yields per filtered cigarette are not related to biomarkers such as carboxyhemoglobin levels, carbon monoxide in exhaled breath and urinary thiocyanate (32, 33). Moreover, for the same nicotine yield/cigarette measured by ISO smoking machines, a large variability in cotinine concentration exists between individuals (34), showing that the “cigarette scale approach” measuring TNCO is misleading both consumers and policy makers.

**Figure 1:**
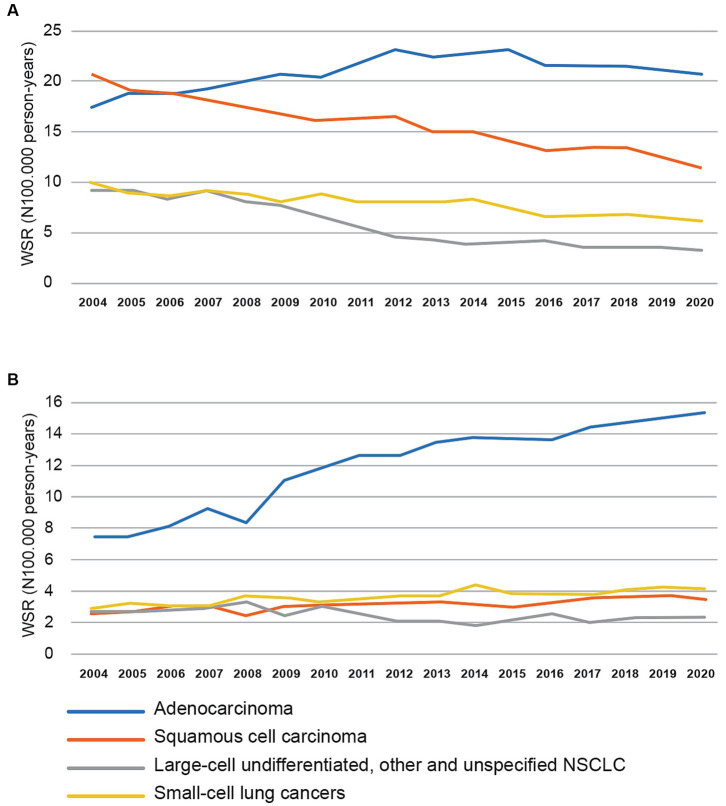
Trends in age-standardized incidence rates (using the World Standard Population) in Belgium from 2004 to 2020 for lung cancer for men **(A)** and women **(B)**. NSCLC = Non Small Cell Lung Cancer. Updated data provided by Belgian Cancer Registry (Brussels, 2023).

In the 1990s, researchers became increasingly aware of the potentially harmful side effects of filtered cigarettes as they seek to explain the alarming increase in lung adenocarcinomas during the 2nd half of the 20th century (35, 36). In 1950, the ratio of lung adenocarcinoma (AD) and squamous cell carcinoma (SQ) was 1:18 in the United States (36). While the incidence of SQ gradually decreased with a decreasing smoking prevalence of unfiltered cigarettes, the incidence of AD increased and exceeded SQ in the US in the 1990s (17). In 2010, the US AD:SQ ratio increased to 1:0.64 in men and 1:0.37 in women (37). Similar trends were also observed in Japan and Europe (38, 39). In 2020, the AD:SQ ratio for Belgian men and women was 1:0.59 and 1:0.25, respectively, (Figure 1). As filter ventilation alters cigarette combustion (longer burn time, lower temperature burn and less complete combustion) (17) and the nitrate content in tobacco blends increased, it was found that more tobacco-specific nitrosamines (TNSAs) are formed, which are more likely to induce peripheral lung AD (35, 36, 40–42). Typical carcinogenic TSNAs present in smoke are 4-(*N*-nitrosomethylamino)-1-(3-pyridyl)-1-butanone (NNK) and *N′*-nitrosonornicotine (NNN) (43). As predicted, compensation to meet nicotine demand appears to be a major contributor in this process: the more intense smoking pattern increased the amount of TSNAs 2- to 3-fold, while deeper inhalation and bigger puffs increased the delivery of TNSAs to the peripheral lungs (35, 36, 41). During the past 20 years, this hypothesis has only been reinforced by new research. Ito et al. (38) examined the relationship between tobacco use and lung cancer histology using tobacco consumption data and population-based incidence data from the US (1973–2005) and Japan (1975–2003). It was revealed that filtered cigarette consumption was positively associated with the incidence of AD, with lag times of 25 and 15 years in Japan and the US, respectively. In contrast, unfiltered cigarette consumption was positively associated with the incidence of SQ, with time lags of 30 and 20 years. Thus, with increasing AD, the average lag time for lung cancer decreased. In 2014, the Surgeon General’s report on smoking and health concluded that the increase in AD was caused by the changing cigarette design. While the evidence was insufficient to specify which changes were responsible, it was indicated that “suggestive evidence” points to ventilated filters (37). In response to this report, Song et al. (17) performed an extensive weight-of-evidence review of both scientific literature and industry documents, leading to the conclusion that filter ventilation strongly contributed to the rise of AD. Increased filter ventilation also increased smoke mutagenicity in *Ames* tests (17). It was suggested that the FDA should consider regulating the use of filters, up to including a ban. These authors also discussed differences in lung cancer histology trends between both sexes. While in the US SQ in men declined since the late 1970s and was surpassed by AD in 1990, it was observed that AD has always been dominant in women and on the rise since 1970. The difference was explained by the fact that American women generally started smoking later in the century and usually smoked filtered cigarettes with lower tar contents (17). Given that the trends from the US are very similar to the incidence rates made available by the Belgian Cancer Registry (Figure 1), we suggest that this explanation also applies to Belgium. It can be concluded that the filter did not protect against lung cancer, but rather contributed to a shift in dominant histology from SQ to AD.

Data on the impact of filtered cigarettes on health effects other than lung cancer are relatively scarce. Some potential hazards such as inhaling cigarette filter fibers are not well studied and the health impact is simply unknown (44). A Chinese case-control study on the impact on oral squamous cell cancer showed overlapping confidence intervals (CI) between smokers of filtered (OR 1.30, 95% CI 1.15–1.48) and unfiltered cigarettes (OR 2.06, 95% CI 1.17–3.62) (45). CIs for filtered cigarettes (OR 2.19, 95% CI 1.19–4.03) and unfiltered cigarettes (OR 3.17, 95% CI 1.50–6.70) were also overlapping for chronic bronchitis in a cross-sectional prevalence study (46). In addition, for coronary heart disease (cohort study) (47) and oral leukoplakia (case-control study) (48), no significant protective effect could be established. Only in a study in subjects with dental implants, a significant increase in marginal bone loss was noticed on the mesial/distal surfaces in unfiltered heavy tobacco smokers (>20 cigarettes/day) (49). After all, the health disadvantage of smoking (both filtered and unfiltered) is much larger in each study, compared to not smoking. More than 70 years after awareness emerged on the causality between tobacco smoking and cancer, further health gains should only be obtained by smoking cessation, prevention and banning. It can be concluded that filtered cigarettes have no proven benefits in preventing adverse health effects of smoking. They create a false sense of security and encourage to smoke more. In that respect, they have been a brilliant marketing tool (6, 11, 15, 16, 19).

## 3. The environmental perspective

While filtered cigarettes have no proven benefits for human health, more than 90% of cigarettes sold worldwide are filtered (44). Globally, 6 trillion cigarettes are produced each year, 5.8 trillion cigarettes are smoked of which 4.5 trillion cigarette butts (CBs) end up in the environment (19, 50). Smoked filters are the most encountered littered item in the world. In Europe, cigarette filters represent 17% of all plastic items and 21% of all single-use plastics (SUPs) counted on beaches (51). In Flanders (northern Belgium), large-scale litter counts at 6,500 locations between 2019–2021 showed that CBs represent 41% of Flemish litter apiece, 2.5% by weight and 1.1% by volume (52). The small size of CBs makes it difficult to recover them during cleaning actions, leaving most butts in the environment. The current filter is a white plug consisting of 12,000 fibers of cellulose acetate, containing TiO_2_ and the plasticizer triacetin (44). Cellulose acetate is a long-lasting material, as its biodegradation ability and rate are reduced with the increasing degree of acetylation, or even suppressed after a substitution degree above 2.5 (53, 54). Throughout the years, CBs undergo different physico-chemical fragmentation processes, leading to the formation of highly persistent microplastics in almost all natural compartments (55), probably threatening human health by entering the food chain (56). Biodegradation is further hampered by microbial nitrogen starvation (57) and the presence of toxic contaminants. As cigarette smoke contains more than 9,500 chemicals (4), a myriad of toxicants (including nicotine) retained by the filter leaches in the environment, stressing aquatic and terrestrial life. Despite the global effects, few studies are available (6). A review of 35 studies has been published by Green et al. (58), indicating that research on terrestrial life is lagging behind. This may be because terrestrial experiments with homogeneous exposure are more difficult to set up than in water.

A systematic review on aquatic organisms was published by Dobaradaran et al. (59), showing high toxicity of CBs impacting survival, growth and reproduction. Smoked filtered cigarette butts with tobacco remnants had higher mortality rates compared to unsmoked filtered cigarette butts for a frog species (*Hymenochirus curtipes*), different fishes (*Clarias gariepinus*, *Atherinops affinis*, *Pimephales promelas*) and tidepool snails (59). It is not surprising that smoked CBs are more toxic than unsmoked, given that the combustion process produces a lot of additional toxic products [e.g., Li and Hecht (4) identified 37 carcinogens in unburned tobacco, which rose to 80 in tobacco smoke]. Crustaceans appear more sensitive than fish, the water flea *Ceriodaphnia dubia* appears to be one of the most sensitive species (60). Recently, ecotoxicological experiments were undertaken in multiple master theses at the University of Antwerp. The amphipod *Grammarus pulex* was exposed by Van Roy (61) to the leachates of freshly collected CBs with tobacco remnants, displaying 96 h-LC_50_ ranges between 0.032–0.059 CB/L. Without tobacco remnants, a 96 h-LC_50_ of 0.1 CB/L was found (62). The pond snail *Lymnaea stagnalis* was studied by Steurbaut (63), exposed to complete CBs (96 h-LC_50_ 0.48 CB/L) and the tobacco fraction of CBs (0.27 CB/L). In a mesocosm experiment, lethal effects were only observed on *Asellus aquaticus* while sublethal effects were detected for the respiration rate of *Corbicula fluminea* (64).

The effects on terrestrial life are less pronounced, but still of concern. Green et al. (65) showed that CBs with filters reduce germination success and shoot lengths of *Lolium perenne* (perennial ryegrass) and *Trifolium repens* (white clover) and alter chorophyll a:b rates. Gill et al. (66) found that CBs may have low toxicity to soil-dwelling invertebrates, as cigarette butt effluent did not impact the survival, growth or feeding of the woodland snail *Aguispira alternata*. Although snails avoided CBs, avoidance decreased within a month along with declining toxicity. Another thesis at the University of Antwerp showed similar results: land snails (*Cornu aspersum*) exposed to print paper soaked in CB leachates showed no mortality or reduction in feeding rate, even at the highest concentration (50 CB/L) (67). Also, some observations have been made on terrestrial vertebrates, including song birds (68, 69). In urban areas, it was noted that some species use CBs in their nests as a repellent against ectoparasites. In both male and female house sparrows (*Passer domesticus*), genotoxic damage in red-blood cells was greater the more CBs were present in the nest.

All these studies show that the ubiquitous presence of toxic cigarette litter is a significant problem for various biota and compartments in different ecosystems. Unfortunately, multiple studies did not distinguish between the effects of the (burned) tobacco rod and the cellulose acetate filter itself, as >90% of the CBs contain a cellulose acetate filter. Therefore, it would be useful to see more ecotoxicological experiments with unfiltered cigarettes in the future.

## 4. The expected effects of a filter ban

Within the framework of single-use plastics, a general ban on cellulose acetate filters would reduce the microplastics burden in the environment. Unfiltered cigarettes thrown into the environment will equally release toxicants [e.g., nicotine, PAHs, VOCs, metals, phthalates (70)] that are a threat for biota. The release will possibly be even more intense but less prolonged. On the other hand, it can be assumed that the shorter “leftovers” will cause only a fraction of the environmental impact of current plastic CBs (71). Given that filters encourage smoking (15, 16), biodegradable filters are not preferred, as they could lead to “greenwashing” for the general population.

Cigarette filters fail to prevent adverse health effects. However, given the gradual shift from SQ to AD since their introduction, a reverse movement may be hypothesized after a filter ban. Both non-small cell lung cancers have a poor prognosis. For Belgian diagnoses between 2015–2020, 5 years survival was 30.2% (95% CI 29.4–30.9%) for AD and 25.1% (95% CI 24.0–26.1%) for SQ (Belgian Cancer Registry). On the other hand, lag times for AD are *ca.* 5 years shorter compared to SQ (38). According to the Belgian Cancer Registry, in 2020 for each histological type, the proportion of cases aged <50 years for AD is almost double that for SQ (men 8.7% vs. 4.9%, women 11.1% vs. 6.5%). As detection and treatment methods are constantly improving and evolving, it is difficult to make an accurate prediction of long-term trends. However, a further decrease in the prevalence of smoking can be expected by banning filters, as unfiltered cigarettes are perceived to be less pleasant, more irritable and unhealthier (11, 16). In a consumer survey in the Netherlands, 12% of the smoking respondents indicated that a filter ban would be a direct reason to quit smoking and to smoke less (71).

The Dutch consumer survey found that support for a filter ban is higher among non-smokers (63%) than smokers (35%) (71). Besides those who would quit or smoke less, 16% would start smoking unfiltered cigarettes and 18% would opt for home-made cigarettes with a reusable filter. Another 6% said they would start using other smoking products such as e-cigarettes, which could potentially lead to an increase in e-waste in the environment. While 27% of respondents were still undecided on their response to a ban, 18% said they would buy filtered cigarettes abroad and 8% illegally on the black market (71). The possibility of purchasing abroad can be largely avoided by implementing the ban at the EU level. The unwanted side effect of filtered cigarettes on the black market, in turn, is a concern for law enforcement and the fight against international criminal networks.

## 5. Public reception and reactions of the tobacco industry

Using these arguments, the SHC proposed a general ban on cigarette filters in April 2023, both on the Belgian and European level (24). As filters only encourage more smoking and give rise to microplastics and toxicants in the environment, it was stated that the filter should be treated as single-use plastics. To achieve maximum social awareness and media coverage, this viewpoint was reviewed and publicly supported by the Belgian Royal Academy of Medicine, the Belgian Society for Medical Oncology, the Belgian Respiratory Society, the Flemish Society of Respiratory Health and Tuberculosis Control, the Walloon Respiratory Fund, the Flemish Institute for Healthy Living, and Domus Medica, the Flemish GP association. The position was widely broadcasted in the national media (newspapers and television) (72) as well as in more specialized medical press (73, 74).

An immediate reaction from Philip Morris Benelux followed, considering the proposal “*unrealistic, ineffective and counterproductive*” (75). According to Philip Morris, the proposal would conflict with the EU’s Tobacco Products Directive, distorting the single EU market and enabling criminal organizations to supply filtered cigarettes. While mainly legal and commercial objections are raised, no attempts were undertaken to disprove the scientific justification of a filter ban. In contrast, Cimabel (Cigarette Manufacturers of Belgium and Luxembourg) stated in a response to the Flemish public-service broadcaster VRT that “*Studies have shown that the lack of a filter leads to an increase in toxins inhaled by consumers. The filter ensures that cigarettes meet the prescribed levels of tar, nicotine and carbon monoxide*” (translated from Dutch) (72). The first argument falls back on the classic “cigarette scale approach” for TNCO, not taking into account compensation behavior and data from human biomonitoring (see Chapter 2). The second argument refers to the ISO smoking machines, which have recently been proven to underestimate the actual exposure of smokers to TNCO and aldehydes (20–23).

## 6. Discussion: how to proceed in a European context?

With the scientific arguments on the table, it is a political choice to introduce a general filter ban. However, the European context is very specific: competences are divided between national member states (including decentralized regional governments) and the European Union, each with its own courts. A recent study ordered by the Dutch government found that the legal feasibility of a ban at the individual member state level is very low, as large adaptions to the Tobacco Products Directive (2014/40/EU) (76) would be needed due to violations of the free movement of goods (Art. 24) (71). This was also highlighted by Philip Morris Benelux (75). Article 7 (7) of the Tobacco Products Directive imposes that member states should prohibit “*the placing on the market of tobacco products with flavourings in any of their components such as filters, papers, packages, capsules or any technical features allowing modification of the smell or taste of the tobacco product concerned or their smoking intensity*.” Further specifying this article, cellulose acetate filters could also be explicitly included under this ban, as they make the smoke more pleasing and induce more smoking. Another, more viable option is the inclusion of a filter ban in the Single-Use Plastics Directive (EU) 2019/904 (77). From 2021, the EU no longer allowed certain single-use plastic items to be placed on the member states market (e.g., plastic straws, stirrers, cutlery plates, cotton bud sticks). Despite cigarette filters being one of the main SUPs found in the environment, they were not included in this ban (15, 18). At the moment, the SUP directive targets reduction of cigarette filters due to marking and labelling requirements, extended producer responsibility and awareness-raising measures (78). Consumers are informed on the presence and effects of plastics in the filters, while tobacco companies should contribute to the cost of the cleaning and collection of filters. However, as cellulose acetate filters do not protect health, it is necessary to rectify this missed opportunity. In a recent letter (April 19th, 2023) from the Dutch Secretary of State for Infrastructure and Water Management to the Dutch Parliament, it is stated that the government is seeking cooperation with other member states to put a ban on filters on the agenda for the next revision of the SUP Directive in 2026 (79). With this initiative, it is our intention to foster this momentum so that policymakers can finally cross the Rubicon treating cigarette filters for what they are: a marketing tool causing global harm. In the meantime, primary prevention remains essential: no smoking should become the norm. In addition, it is known that adolescents and young adults who are aware of filters’ environmental harm are more supportive of cigarettes sales bans (80). Therefore, specific education is needed on the environmental aspects of cigarette filters and microplastics among these groups.

## Data availability statement

The original contributions presented in the study are included in the article/supplementary material, further inquiries can be directed to the corresponding authors.

## Author contributions

SE: Conceptualization, Project administration, Writing – original draft, Investigation. GS: Investigation, Supervision, Writing – original draft. FL: Investigation, Writing – original draft. AJ: Investigation, Writing – original draft. NL: Investigation, Writing – original draft. J-MR: Investigation, Writing – original draft. LB: Investigation, Writing – original draft. PS: Investigation, Supervision, Writing – original draft.
